# Hierarchical on-surface assembly of nanoribbons through concurrent hydrogen- and chalcogen-bonding interactions

**DOI:** 10.1039/d5sc08883f

**Published:** 2026-05-20

**Authors:** Antonio Caporale, Luca Persichetti, Gabriele Anselmi, John Lloyd Gildo, Conor Hogan, Deborah Romito, Davide Bonifazi, Luca Camilli

**Affiliations:** a Dipartimento di Fisica, Università degli studi di Roma Tor Vergata Rome Italy luca.camilli@uniroma2.eu; b CNR-Istituto di Struttura della Materia (CNR-ISM) Roma Italy; c Department of Organic Chemistry, Faculty of Chemistry, University of Vienna Vienna Austria davide.bonifazi@univie.ac.at

## Abstract

Secondary Bonding Interactions (SBIs), particularly chalcogen bonding interactions (ChBIs), offer powerful opportunities to direct the assembly of functional organic materials on surfaces. Here we combine Te-based ChBIs with F⋯H hydrogen bonds (HBs) to drive the hierarchical engineering of supramolecular nanoribbons on Au(111) using a chalcogenazolo-pyridine derivative. Low-temperature scanning tunnelling microscopy imaging reveals that the molecules first undergo directional Te⋯N chalcogen-bonded dimerisation, followed by HB-mediated polymerisation into robust nanoribbon architectures. Density functional theory calculations confirm the adsorption geometries and intermolecular binding modes. The Au(111) herringbone reconstruction templates the nanoribbon orientation and maximum attainable length, with the face-centred cubic regions being the preferred adsorption sites. Scanning tunnelling spectroscopy reveals two intrinsic electronic fingerprints at +0.6 V and +1.8 V, corresponding to the LUMO and LUMO+1 of the dimeric repeat unit, respectively. The d*I*/d*V* mapping visualises their distinct spatial distributions along the ribbon backbone and edges. Control experiments with a non-pyridyl congener that cannot engage in Te⋯N ChBIs yield only simple linear assemblies, confirming the pivotal role of ChBIs in enabling hierarchical ordering.

## Abstract 2

We report on the bottom-up construction of supramolecular chalcogen-bonded nanoribbons with well-defined electronic fingerprints built entirely from directional non-covalent interactions.

## Graphical abstract

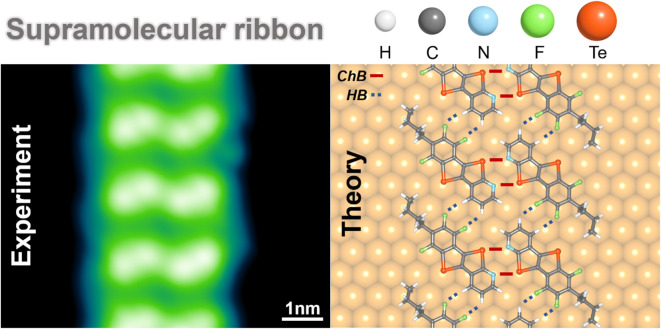

## Introduction

Over the past two decades, various noncovalent interactions have been successfully employed to control the supramolecular assembly of functional organic molecules on surfaces.^1–3^ H-bonding, halogen-bonding, and π–π interactions have proven particularly effective in guiding these assemblies, especially on surfaces.^4–7^ Within this context, Secondary Bonding Interactions (SBIs) have garnered increasing attention.^8^ Unlike purely electrostatic or dispersion-driven contacts, SBIs feature a significant orbital contribution, typically described as n_Y_ → σ_E–X_ donation. This orbital-mixing component not only enhances the strength and directionality of the interaction but also directly impacts charge delocalisation, a key factor for optoelectronic applications.^9^ In particular, chalcogen bonding interactions (ChBIs) have emerged as a versatile means for molecular recognition^10–14^ and, more specifically, for organising molecules in the solid state.^13,15–18^ Formed when heavier chalcogen atoms such as Se or Te are used, ChBIs combine van der Waals, electrostatic (*i.e.*, σ-hole), steric and orbital-mixing contributions, enabling the construction of robust supramolecular architectures.^14,19,20^ Recently, our groups demonstrated for the first time the on-surface assembly of pyrenyl derivatives containing chalcogenazolo-pyridine (CGP) units through double directional Ch⋯N chalcogen bond interactions involving Te- or Se-atoms.^21,22^ Specifically, scanning tunnelling microscopy (STM) imaging showed that the molecules undergo noncovalent chiral dimerisation on metal surfaces. However, realising truly supramolecular chalcogen-bonded materials, and ultimately functional materials, requires demonstrating that ChBIs are robust and directional enough to template extended non-covalent architectures, and not only discrete dimers as shown so far.^21^ With this aim, we build on these results to show that 7-butyl-6,8,9-trifluorobenzo[4′,5′]telluropheno[2′,3′:4,5]telluropheno[2,3-β]pyridine (3FBP-2Te), previously used to prepare supramolecular chalcogenide semiconducting materials in the solid state,^23^ can undergo molecular recognition and form hierarchical supramolecular chalcogenide nanoribbons on Au(111). These ribbons are held together by concurrent ChBIs and F⋯H hydrogen bonds (HBs). Notably, low-temperature scanning tunnelling microscopy (LT-STM) experiments reveal substrate-templated growth, whereas scanning tunnelling spectroscopy data unveil distinct electronic states of the nanoribbons that remain unchanged as a function of the ribbon length. To the best of our knowledge, the nanoribbons reported here provide this proof of principle, establishing that Te-based chalcogen bonds can drive hierarchical assembly beyond the pairwise level.

## Methods

The molecules studied here were sublimated in ultrahigh vacuum from a commercial evaporator (Kentax) onto a Au(111) single crystal that had been cleaned by standard Ar^+^ sputter/anneal cycles. During sublimation, the Au(111) substrate was kept at room temperature, with the pressure in the chamber being below 5 × 10^−10^ mbar. All STM measurements were performed using a commercial Infinity system from Scienta Omicron held at 10 K. All experimental images were analyzed using the Gwyddion software.^24^ The STM images were calibrated so that the measured Au lattice constant would coincide with the one from the simulation after geometry optimization (lattice constant: 4.12 Å). d*I*/d*V* spectra were collected with lock-in frequency 653 Hz and ac voltage modulation 23 mV. Density functional theory (DFT) calculations follow the recipe reported in ref. 21 and are described in detail in the SI.

## Results and discussion

**Fig. 1:**
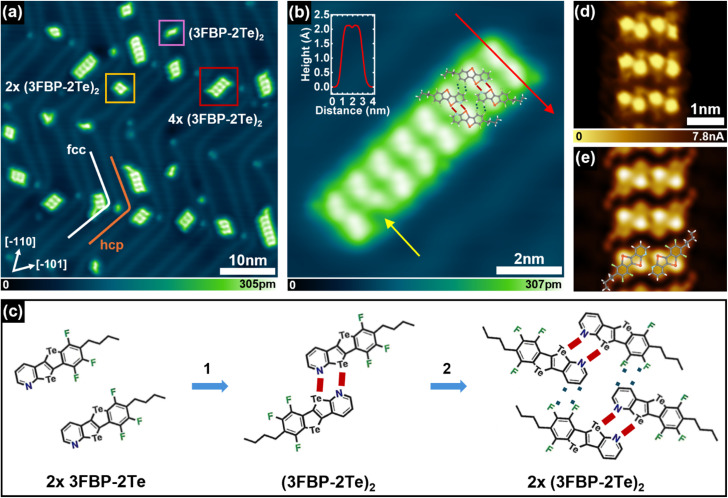
3FBP-2Te molecules deposited with submonolayer coverage on Au(111) kept at room temperature. (a) STM topographic image of ribbons with different lengths on Au(111). In the purple square, a dimeric structure, (3FBP-2Te)_2_ is highlighted. In the yellow and red boxes, double-unit (*n* = 2) and four-unit (*n* = 4) ribbons are displayed, respectively. (b) High-resolution STM image of octameric ribbon [(3FBP-2Te)_2_]_8_. Inset: line profiles across the solid red arrow. (a) and (b) have the same crystallographic orientation. (c) Proposed hierarchical self-assembly mechanism on the Au(111) surface: ChBs drive the dimerization into (3FBP-2Te)_2_, while HBs link dimers into ribbons [(3FBP-2Te)_2_]*_n_* of different lengths (*n*). (d) Experimental constant-height STM image acquired with a CO-functionalized tip on a ribbon. (e) Simulated STM image with overlaid the DFT-optimized model of a dimer. Imaging parameters: (a) tunnelling current, *I*_t_ = 25 pA; bias voltage, *V*_b_ = 0.1 V. (b) *I*_t_ = 85 pA; *V*_b_ = 0.1 V. (d) *V*_b_ = 5 mV. (e) Simulated constant current image at *V*_b_ = 10 mV.

The archetypal molecule 3FBP-2Te was synthesised following the previously reported literature protocol by our group.^23^ When sublimated in vacuum onto Au(111) kept at room temperature (RT), 3FBP-2Te undergoes oligomerisation and assembles into nanoribbon-like supramolecular polymers of variable length (Fig. 1a). It can be noted that the ribbons preferably lie on the face-centred-cubic (fcc) domains of the well-known (22 × √3) herringbone reconstruction of the Au(111) surface, which consists of a periodic array of fcc and hexagonal-close-packed (hcp) domains separated by ridges where atoms fall out of registry.^25,26^ The coloured boxes in Fig. 1a pinpoint ribbons of different lengths. A single, non-assembled, molecular module is highlighted in purple. The yellow and red boxes instead identify ribbons of lengths of two and four molecular modules, respectively. A close-up view of one of these ribbons (Fig. 1b) reveals that its fundamental unit appears to be constituted of two molecules (see also the line profile across the red arrow shown in the inset of Fig. 1b). The shape and measured length of 2.1 nm align with two 3FBP-2Te monomers forming a dimeric structure (3FBP-2Te)_2_ (purple box in Fig. 1a), where they are mirrored and shifted so that the Te atoms of one molecule face the *N*-pyridyl atoms of the other one, as represented by the ball-and-stick model overlaid on the ribbon in Fig. 1b and obtained by density functional theory (DFT) calculations after geometry optimization (see below). This recognition pattern is common for CGP moieties on metal surfaces.^21^ These dimers then further assemble into ribbons ([(3FBP-2Te)_2_]*_n_*)*via* H-bonds between two fluorine atoms on one benzene ring and two aromatic hydrogen atoms of an adjacent dimer. A schematic of the proposed hierarchical assembly mechanism is shown in Fig. 1c. Our interpretation is supported by the comparison between the experimental STM image obtained in constant-height mode with a CO-functionalized tip and shown in Fig. 1d and the simulated STM image obtained by DFT and displayed in Fig. 1e. It is worth noting that the concurrent presence of ChBIs and HBIs makes the formation of the self-assembled ribbons robust against the presence of defects, as the presence of structural imperfections does not seem to affect their overall integrity. Indeed, as exemplified by the octameric ribbon displayed in Fig. 1b, even when one of the (3FBP-2Te)_2_ that make the fundamental unit of the ribbon is defective (see yellow arrow in Fig. 1b), the overall integrity of the ribbon is seemingly unaltered. In this specific case, even though not all the H-bonds can be formed between the two (3FBP-2Te)_2_ adjacent to the defective one, their geometrical conformation is not affected because the individual 3FBP-2Te monomers within the dimers are still locked in their position owing to the ChBIs.

**Fig. 2:**
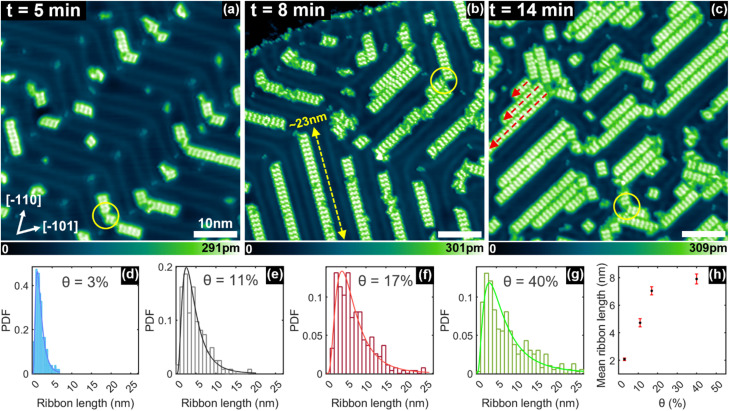
3FBP-2Te molecules deposited at different submonolayer coverages on Au(111) kept at room temperature. (a–c) STM topographic images of self-assembled ribbons on Au(111) after a sublimation time (*t*) of 5 min, 8 min, and 14 min, respectively, and corresponding to coverages, *θ*, of 11%, 17% and 40%. (d–g) PDFs of the length of ribbons at coverages, *θ*, of 3%, 11%, 17% and 40%, respectively. The histogram data are obtained from STM images. The continuous lines in each panel are a lognormal fit to the data. (h) Mean length of the ribbons extracted from the lognormal fit of the PDFs in (d)–(g) and plotted as a function of the coverage, *θ*. (a–c) share the same lateral scale and crystallographic orientation. Imaging parameters: *I*_t_ = 25 pA; *V*_b_ = 0.1 V.

Fig. 2a–c shows STM topographic images taken on the same sample at increasing sublimation times. As expected, both surface coverage and ribbon length increase with longer sublimation periods. Initially, ribbons are confined to the fcc regions of the Au(111) reconstruction (Fig. 2a). Their presence, however, modifies the herringbone reconstruction so that, at higher coverages, the fcc domains increase in size in order to accommodate neighbouring ribbons (Fig. 2b and c). The zigzag pattern of the herringbone reconstruction eventually limit ribbons' growth at longer sublimation times, as exemplified by the red dashed arrows in Fig. 2c. For example, when the underlying herringbone pattern is straight, the ribbon length can reach tens of nanometres (see the yellow arrow in Fig. 2b). Interestingly, at the turning points of the Au(111) reconstruction, also known as herringbone “elbows”, it is common to observe two ribbons forming a 120° angle, as highlighted by the yellow circles in Fig. 2. This angle is dictated by the pattern of the herringbone reconstruction, thereby proving again the templating role of the Au(111) surface.

Ribbon length distribution was analysed for different surface coverages (*θ*) by calculating their probability density functions (PDFs), as shown in Fig. 2d–g. Since this is a case of progressive growth, the ribbon length distribution can be described by the Fokker–Planck equation, whose solution is the lognormal distribution.^27^ This distribution has been used here for fitting the experimental data (solid curves in Fig. 2d–g). From these fits, the mean ribbon length is determined and plotted against surface coverage in Fig. 2h. After a linear increase for coverages below 20%, the mean length reaches a plateau due to the constraints imposed by the zig-zag pattern of the herringbone reconstruction, as discussed above. Incidentally, ribbons formed on highly oriented pyrolytic graphite, which does not have a reconstructed surface, appear to be straight and parallel to each other (Fig. S1 in SI).

**Fig. 3:**
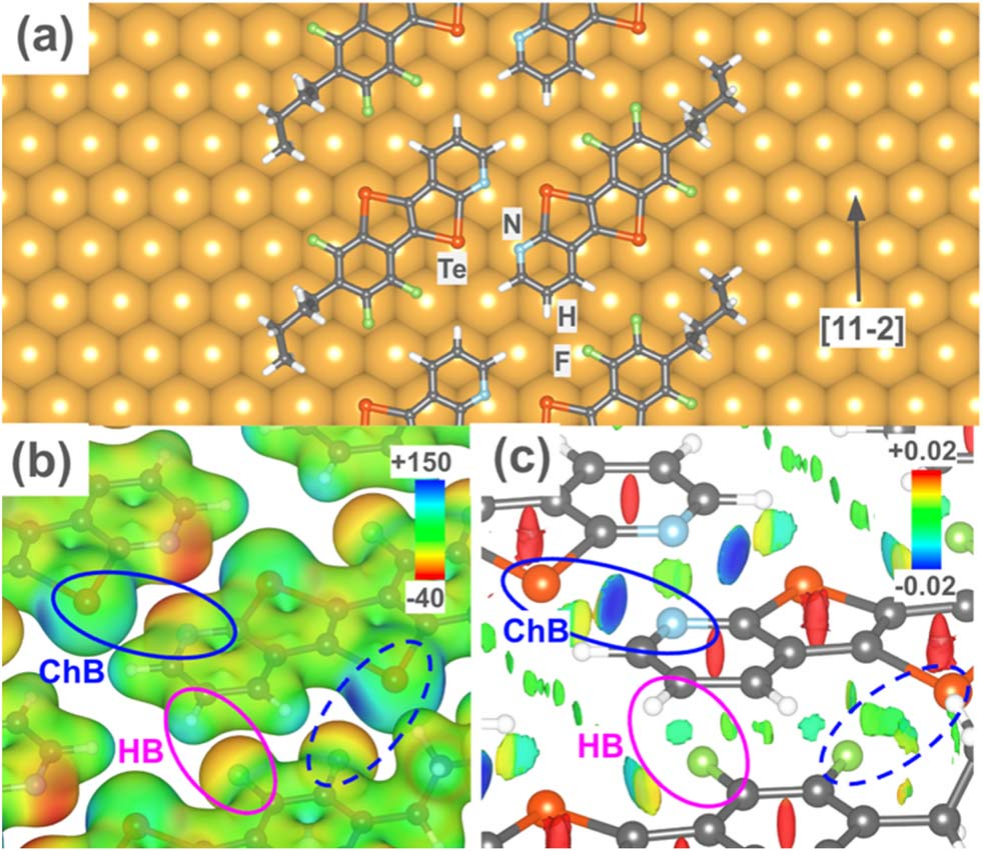
DFT calculations of the [(3FBP-2Te)_2_]*_n_* ribbon on Au(111). (a) Adsorption geometry. (b) Electrostatic potential (in au) superimposed on a charge density isosurface (*ρ* = 0.025 au). (c) Reduced density gradient (on the 0.5 au isosurface): blue and red regions indicate attractive and repulsive interactions respectively, green indicates weak (*e.g.* van der Waals) interactions. In (b) and (c) the substrate has been removed for clarity. The Te⋯N, H⋯F, and Te⋯F bonding interactions are indicated.

DFT calculations confirm the proposed binding mechanisms and ribbon geometry on Au(111). As shown in Fig. 3a, the 3FBP-2Te moieties adsorb on Au(111) in a planar geometry with both Te atoms in on-top positions. This alignment favours formation of [(3FBP-2Te)_2_]*_n_* ribbons along the [11−2] direction. For simplicity, we analyse the intermolecular binding energies of freestanding planar (3FBP-2Te)_2_ dimers and ribbons (Fig. S2 in SI), as well as the ribbons in their adsorbed geometry (*i.e.* with the substrate removed). We find that the Te⋯N ChBIs are over twice as strong as the F⋯H HBs (−11.83 kcal mol^−1^ and −4.33 kcal mol^−1^ respectively; see Table S1 in SI). Analysis of the electrostatic potential (Fig. 3b) and reduced density gradient (Fig. 3c) in the adsorbed ribbon geometry confirm the presence of secondary bonding interactions. We also note the presence of multiple weaker interactions within the ribbon assembly, including between Te and F (dashed oval). Upon adsorption, the optimal Te⋯N and F⋯H bond lengths are only slightly increased, with concomitant lowering of the total ribbon binding energy from −22.82 kcal mol^−1^ to −17.10 kcal mol^−1^; see SI for more details.

**Fig. 4:**
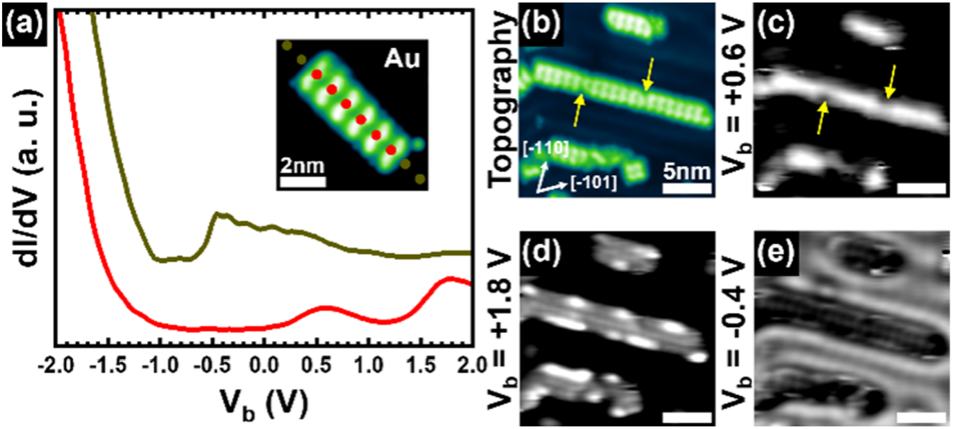
Electronic properties of [(3FBP-2Te)_2_]_6_ ribbons. (a) Averaged d*I*/d*V* spectra collected on the locations marked by the coloured dots in the STM topographic image reported in the inset. (b) STM topographic image and (c–e) corresponding constant-current d*I*/d*V* maps collected at a bias voltage of: *V*_b_ = +0.6 V, +1.8 V, and −0.4 V, respectively. The four images share the same lateral scale. Imaging parameters: (a) *I*_t_ = 50 pA; *V*_b_ = 2.0 V. (b) *I*_t_ = 50 pA; *V*_b_ = 0.1 V. (c) *I*_t_ = 50 pA. (d) *I*_t_ = 50 pA.

Next, we probed the electronic structure of an individual hexameric ribbon [(3FBP-2Te)_2_]_6_ using lock-in-based scanning tunnelling spectroscopy (STS). Fig. 4a shows averaged d*I*/d*V* spectra acquired either along the ribbon (red curve) or on the Au(111) surface (olive green curve) at the locations marked by the coloured dots in the inset STM image. The individual curves are reported in SI for clarity (Fig. S5). Bare Au(111) exhibits its characteristic Shockley surface state peaked at around −0.4 V and a rapid rise in the density of states at voltages below −1.0 V. In the conduction band, the density of states does not display noteworthy features within the studied energy range. The average spectrum collected on the ribbon instead reveals two characteristic spectroscopic fingerprints, located at +0.6 V and +1.8 V, respectively. It is difficult to comment on the rise in the density of states at voltages below −1.0 V because of the strong contribution of the valence band of the underlying Au(111).

To further characterise the electronic properties of the system, we acquired constant-current d*I*/d*V* maps of the ribbon shown in Fig. 4b at bias voltages (*V*_b_) corresponding to the well-defined peaks observed in the d*I*/d*V* spectra of the ribbon in Fig. 4a, that is at +0.6 V and +1.8 V. For comparison, we also collected a constant-current d*I*/d*V* map at the energy position of the Au(111) surface state (that is −0.4 V). These maps are displayed in Fig. 4c–e. The first observation is that the electronic states at +0.6 V and +1.8 V indeed belong to the ribbons, as the Au(111) surface appears dark in the corresponding maps in Fig. 4c and d. The second observation is that the electronic state at +0.6 V is found continuously along the middle of the ribbons, whereas the one at +1.8 V is spatially confined to the edges, with an alternating nodal structure. Their consistency among all the imaged ribbons, which exhibit different lengths, suggests that the two electronic states are, respectively, the lowest unoccupied molecular orbital (LUMO) and the LUMO+1 of the dimeric molecular unit (3FBP-2Te)_2_. This observation is consistent with the calculated spatial distribution of the LUMO and LUMO+1 of isolated (3FBP-2Te)_2_ dimers (Fig. S4 in SI). Lastly, it is worth noting that defects attenuate the LUMO intensity (see the region indicated by the yellow arrows in Fig. 4b and c), while leaving the LUMO+1 unaffected. As expected, there is no signal coming from the ribbons in the reference d*I*/d*V* map acquired at −0.4 V, which corresponds to the Au(111) surface state (Fig. 4e).

**Fig. 5:**
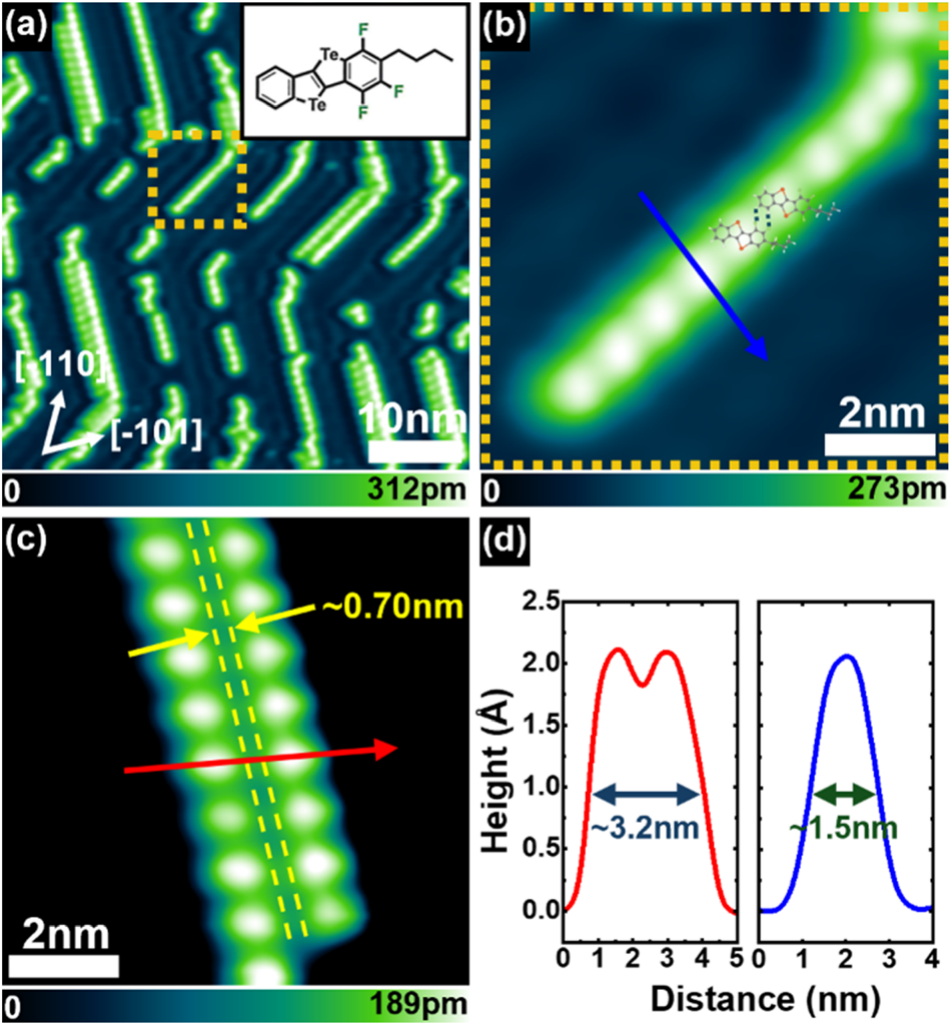
Benzo-3FBP-2Te molecules deposited at submonolayer coverage on Au(111) kept at room temperature. (a) STM topographic image of linear assemblies on Au(111), where the single unit is a Benzo-3FBP-Te molecule. (b) STM topographic image of a detail of a chain. (c) Two adjacent assemblies. (d) Line profiles across the solid blue and red arrows in (b) and (c), respectively. All the images share the same crystallographic orientation. Imaging parameters: (a–c) *I*_t_ = 50 pA; *V*_b_ = 0.1 V.

As a control experiment, we investigated molecular congeners in which the pyridyl N-atom is substituted with a C–H group, namely Benzo-3FBP-2Te (inset of Fig. 5a). The absence of N-atoms in Benzo-3FBP-2Te is confirmed by X-ray photoemission spectroscopy (*cf.* Fig. S6 and S7 in SI). Upon sublimation in vacuum onto Au(111) at RT, we observed the formation of linear assemblies (Fig. 5a), which predominantly localise within the fcc regions of the herringbone reconstruction, similar to the behaviour observed for 3FBP-2Te. In this case, however, the assembly unit consists of only one molecule of Benzo-3FBP-Te, as shown in a magnified view of such a chain in Fig. 5b. The line profile, taken along the solid blue line and displayed in the right panel of Fig. 5d, confirms the presence of a single molecule that acts as the assembly's building block. Here, replacing the pyridine ring with an aryl ring results in a molecule that lacks *N*-pyridyl atoms and therefore cannot form N⋯Te ChBI. Consequently, the observed assemblies arise from the stacking of individual Benzo-3FBP-Te monomers, mainly driven by intermolecular F⋯H interactions. The ball-and-stick model, overlaid on the STM image, supports our interpretation. DFT calculations of Benzo-3FBP-Te adsorption and intermolecular binding are reported in the SI (see Table S1 and Fig. S3).

Interestingly, some of the linear assemblies in Fig. 5a form closely spaced pairs. A close-up view of such a structure is reported in Fig. 5c. The line profile along the red arrow (Fig. 5d, left) reveals that these parallel assemblies maintain significant separation between themselves. Notably, the interchain distance markedly exceeds that observed for ChB-stabilised dimers in Fig. 1b, providing further evidence that assemblies of Benzo-3FBP-2Te lack ChBIs, and thus are not hierarchically organised.

## Conclusions

In conclusion, our study demonstrates hierarchical molecular assembly on Au(111) *via* concurrent chalcogen and hydrogen-bonding interactions. Using Te-containing chalcogenazolo-pyridine moieties, we achieved both dimerisation *via* directional Te⋯N ChBs and ribbon elongation through F⋯H hydrogen bonds between dimers. The Au(111) herringbone reconstruction templates the ribbon orientation and length, with fcc domains of the Au(111) surface being preferred adsorption regions. DFT calculations support our interpretations of the ribbon geometries and binding modes. STS performed on individual nanoribbons reveals the presence of the LUMO and LUMO+1 states within the probed energy range. By performing d*I*/d*V* maps, we manage to visualise the spatial distribution of these two frontier orbital states. Control experiments with non-pyridyl analogues yield linear assemblies lacking ChBI contributions, in which the building blocks are held together by HBs. By extending chalcogen-bond-mediated assembly from isolated dimers to extended one-dimensional nanoribbons with well-defined electronic fingerprints, this work represents a step toward the bottom-up construction of supramolecular chalcogen-bonded semiconductors built entirely from directional non-covalent interactions.

## Author contributions

AC, GA: investigation and formal analysis; AC, LP, LC: methodology; DR: molecular synthesis; JLG, CH: simulations; DB: conceptualisation; LC: supervision; DB, LC: funding acquisition; AC, DB, LC, CH: writing – original and revised draft; all authors: writing – review and editing.

## Conflicts of interest

There are no conflicts to declare.

## Supplementary Material

SC-017-D5SC08883F-s001

## Data Availability

The data supporting the findings of this study are openly available in the Zenodo repository at https://doi.org/10.5281/zenodo.17582722. Supplementary information (SI): STM experiments on HOPG; X-ray photoemission spectroscopy experiments; individual d*I*/d*V* spectra, DFT calculations and NMR analysis. See DOI: https://doi.org/10.1039/d5sc08883f.
