# Supplementary material for: Hierarchical on-surface assembly of nanoribbons through concurrent hydrogen- and chalcogen-bonding interactions
Source: Chem Sci. 2026 May 20;17(26):13148–54. doi: 10.1039/d5sc08883f (PMC13215391; doi:10.1039/d5sc08883f)
Supplement: SC-017-D5SC08883F-s001 [file SC-017-D5SC08883F-s001.pdf]

Supplementary Information (SI) for Chemical Science.
This journal is © The Royal Society of Chemistry 2026

Supplementary Information for

On-surface assembly of nanoribbons through concurrent hydrogen- and chalcogen-bonding interactions

A. Caporale[a], L. Persichetti[a], Gabriele Anselmi,[a] John Lloyd Gildo,[a] Conor Hogan,[a,b] D. Romito[c], D. Bonifazi[c] and L. Camilli[a]

a Department of Physics, University of Rome "Tor Vergata", 00133 Roma, Italy;

b CNR-Istituto di Struttura della Materia (CNR-ISM), Roma, Italy

c Department of Organic Chemistry,Faculty of Chemistry, University of Vienna, 1090 Vienna, Austria

STM experiments of 3FBP-2Te/HOPG

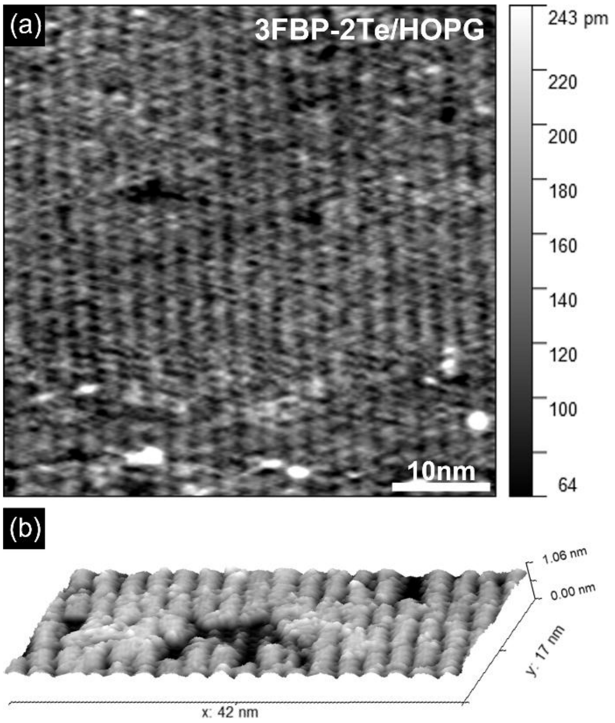

Figure S1 – **3FBP-2Te** molecules deposited on highly oriented pyrolytic graphite (HOPG). HOPG was kept at room temperature during molecule evaporation in vacuum. (a) STM topographic image of parallel ribbons on HOPG whose length exceeds tens of nm. (b) A 3D close-up view of the ribbons on HOPG. Imaging parameters: (a) I_t = 50 pA; V_b = 2.66 V. (b) I_t = 50 pA; V_b = 2.64 V.

DFT calculations

Methodology: Calculations were performed using the quantum-ESPRESSO code[1] in a standard planewave (cutoff 45/360 Ry) and pseudopotential (ultrasoft RRJK) scheme. The PBE exchange correlation functional[2] was used along with the Grimme-D3-BJ van der Waals correction[3]. Isolated dimers were computed in a 45x35x25 $Å^3$ cell; freestanding ribbons were computed using the vc-relax method in a 45xDx25 $Å^3$ cell where the ribbon period D is optimized (D = 9.65 Å for **(3FBP-2Te)$_2$**, 9.58 Å for **Benzo-3FBP-2Te**. For the surface calculations, a four-layer Au(111) slab was used (a_0 = 4.12 Å) with the backmost two layers fixed. The supercell size was 46.64x10.10x32.98 $Å^3$**.** A 1x8x1 k-point sampling was used for surface/ribbon calculations; gamma point sampling was used otherwise. Geometry optimizations used a tight 5 meV/Å threshold. To mimic the formation of **3FBP-2Te** dimers and ribbons on the surface, we first performed geometry optimizations in which Te, N, and F atoms were constrained to lie in the same plane. This allowed us to disentangle different ChB and HB interactions. For true on-surface calculations, we assumed that Te atoms adsorb on Au(111) in on-top positions, following our previous work with CGP moieties[4], and thereafter allowed unconstrained relaxation. From the freestanding studies we noticed that the freestanding ribbon period D matches well with the lattice periodicity along the [11-2] direction. As shown in Table S1, the Te⋯N bond length is increased by 0.1 Å upon adsorption while the F⋯H bonds differ by 0.1-0.2 Å. Note that the imposed periodicity also forces each dimer in the ribbon to align in parallel: as a result, the F⋯Te distance

is stretched by 0.6 Å with respect to the dimer geometry in freestanding configuration. Molecular electrostatic maps (ρ = 0.025 au) and reduced density gradients (RDG) were analysed using VESTA[5]. RDG isosurfaces are colorized using the product of the charge density and sign of the second eigenvalue of the electron density Hessian matrix in the range [-0.02:0.02] au[6]. STM images were computed in constant current mode using the Tersoff-Hamann approximation[7].

	**Geometry**	**E_b (eV)**	**E_b (kcal/mol)**	**Te-N (Å)**	**F-H (Å)**	**F-Te (Å)**
**$(3FBP\text{-}2Te)_2$**	ChBl dimer	-0.513	-11.828	2.90, 2.90	-	-
	HB dimer	-0.188	-4.327	-	2.83, 2.43	3.33
	Ribbon (free)	-0.989	-22.814	2.90, 2.90	2.50, 2.53	3.43
	Ribbon (on Au)	-0.741	-17.099	2.99,3.00	2.60, 2.58	3.93
**$(Benzo\text{-}3FBP\text{-}2Te)_2$**	HB dimer	-0.167	-3.853	-	2.47, 2.80	3.33
	Ribbon (free)	-0.171	-3.955	-	2.54,2.67	3.37
	Ribbon (on Au)	-0.059	-1.365	-	2.61, 2.70	3.93

Table S1 – Binding energies E_b (in eV and kcal/mol) of **$(3FBP\text{-}2Te)_2$** and **$(Benzo\text{-}3FBP\text{-}2Te)_2$** dimers and ribbons (in ChB and HB interaction geometries, see Figure S2 below), and key interatomic distances (in Å, for both bonds if present). Ribbon (on Au) indicates the adsorbed geometry with the Au substrate removed. Binding energies are calculated relative to the freely relaxed, unconstrained molecule.

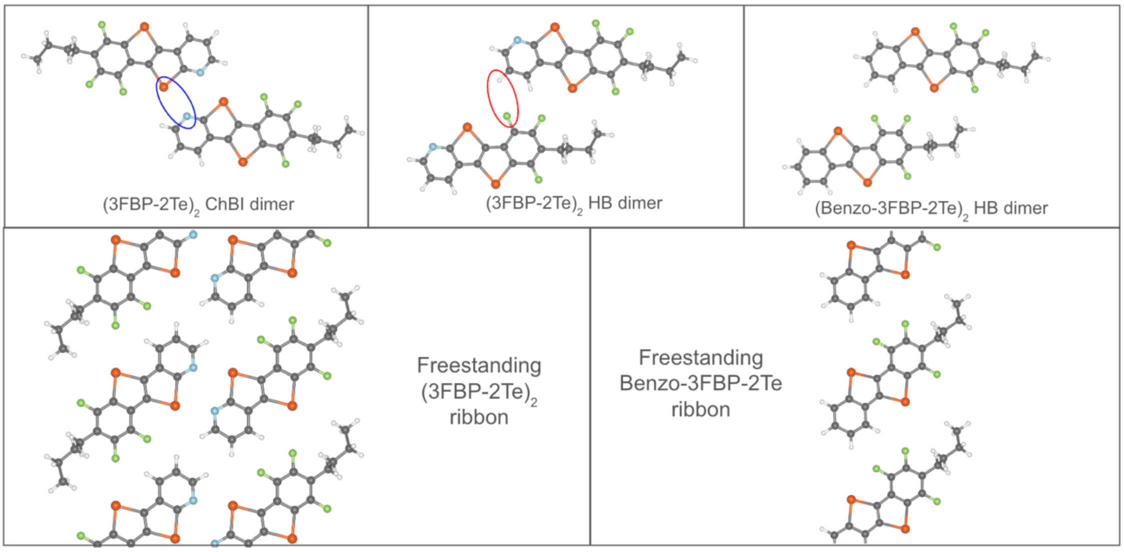

Figure S2 – Optimized geometries (PBE-D3BJ) for freestanding dimers (top) used to isolate ChB and HB interaction energies present in the ribbon geometries (bottom). Top views are shown.

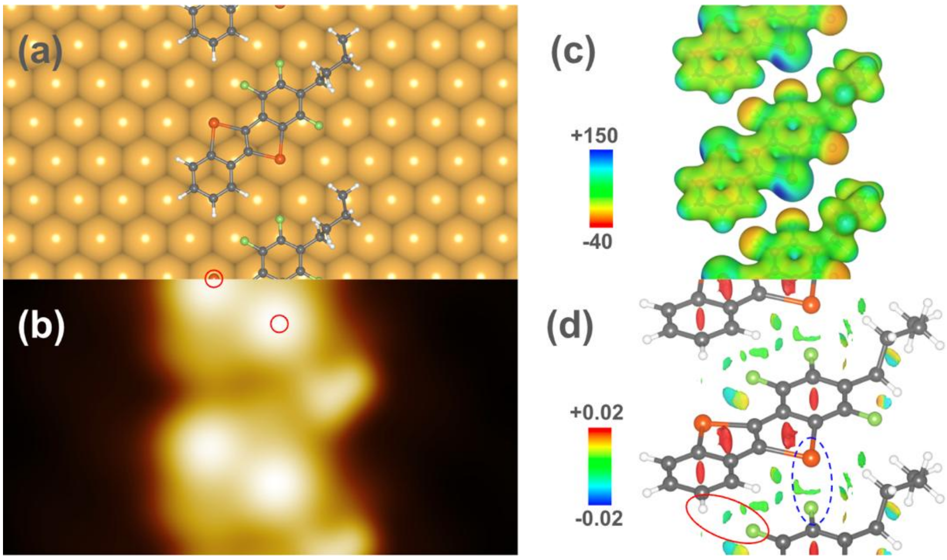

Figure S3 – DFT calculations of **Benzo-3FBP-2Te** assembly on Au(111). (a) Adsorption geometry. (b) Simulated STM image at constant current, V_b = 0.1eV. (c) Electrostatic potential (in au) superimposed on a charge density isosurface (ρ = 0.025 au). (d) Reduced density gradient (on the 0.5 au isosurface): blue and red regions indicate attractive and repulsive interactions respectively, green indicates weak (e.g. van der Waals) interactions. In (c) and (d) the substrate has been removed for clarity; HBs (red oval) and Te···F interactions (dashed blue oval) are indicated.

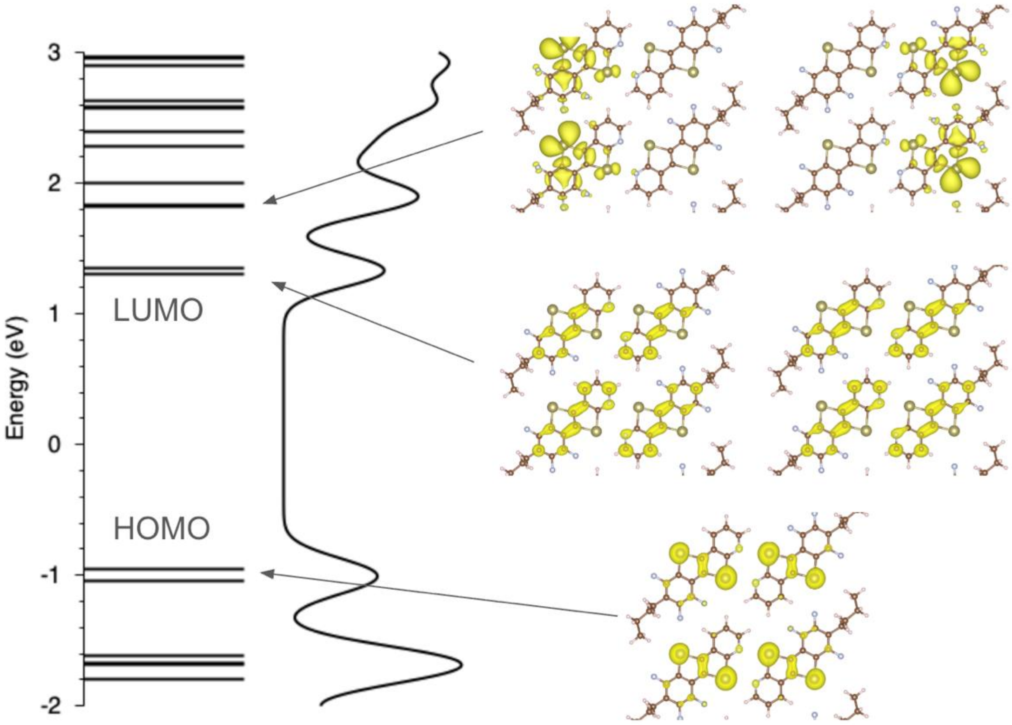

Figure S4 – The density of states (left) and frontier orbitals of the freestanding **Benzo-3FBP-2Te** assembly (right).

STS of (3FBP-2Te)$_2$]$_6$ on Au(111)

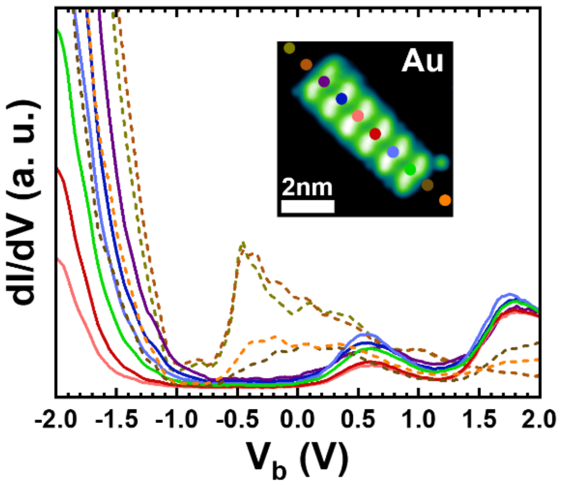

Figure S5 – Individual dI/dV spectra of the hexameric ribbon **[(3FBP-2Te)$_2$]$_6$** reported in Fig. 4a in the main text, collected on the locations marked by the coloured dots in the scanning tunnelling microscopy image reported here in the inset. Lock-in frequency 653Hz, AC voltage modulation 23 mV.

XPS analysis

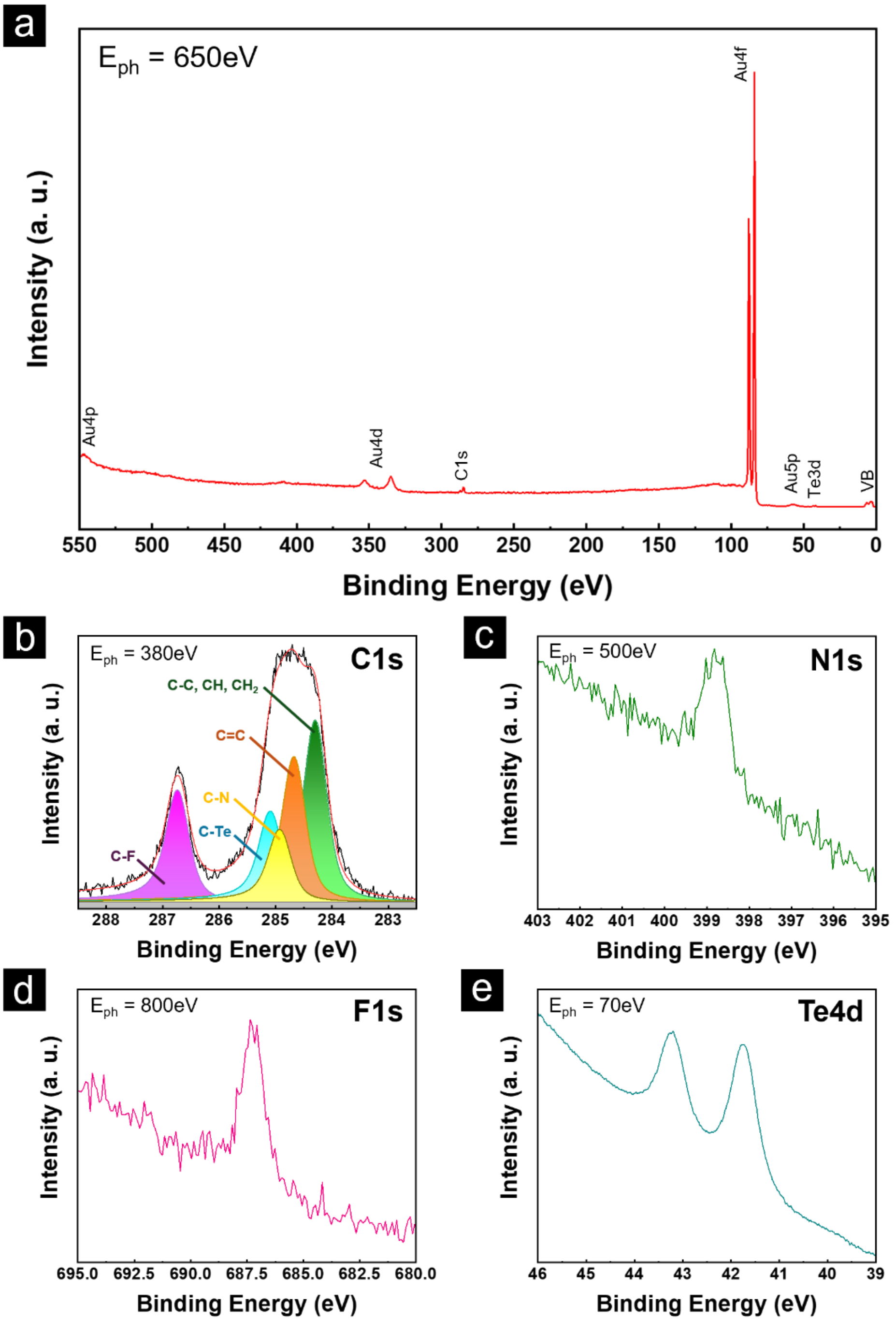

Figure S6 – X-ray photoemission spectroscopy (XPS) data collected on an Au(111) sample after deposition of 3FBP-2Te molecule. (a) Survey with labelled XPS peaks; (b) C 1s core level fitted with five components indicated as C=C, (C-C, C-H, C-H_2), C-Te, C-N, C-F; (c) N 1s core level; (d) F 1s core level; (e) Te 4d core level.

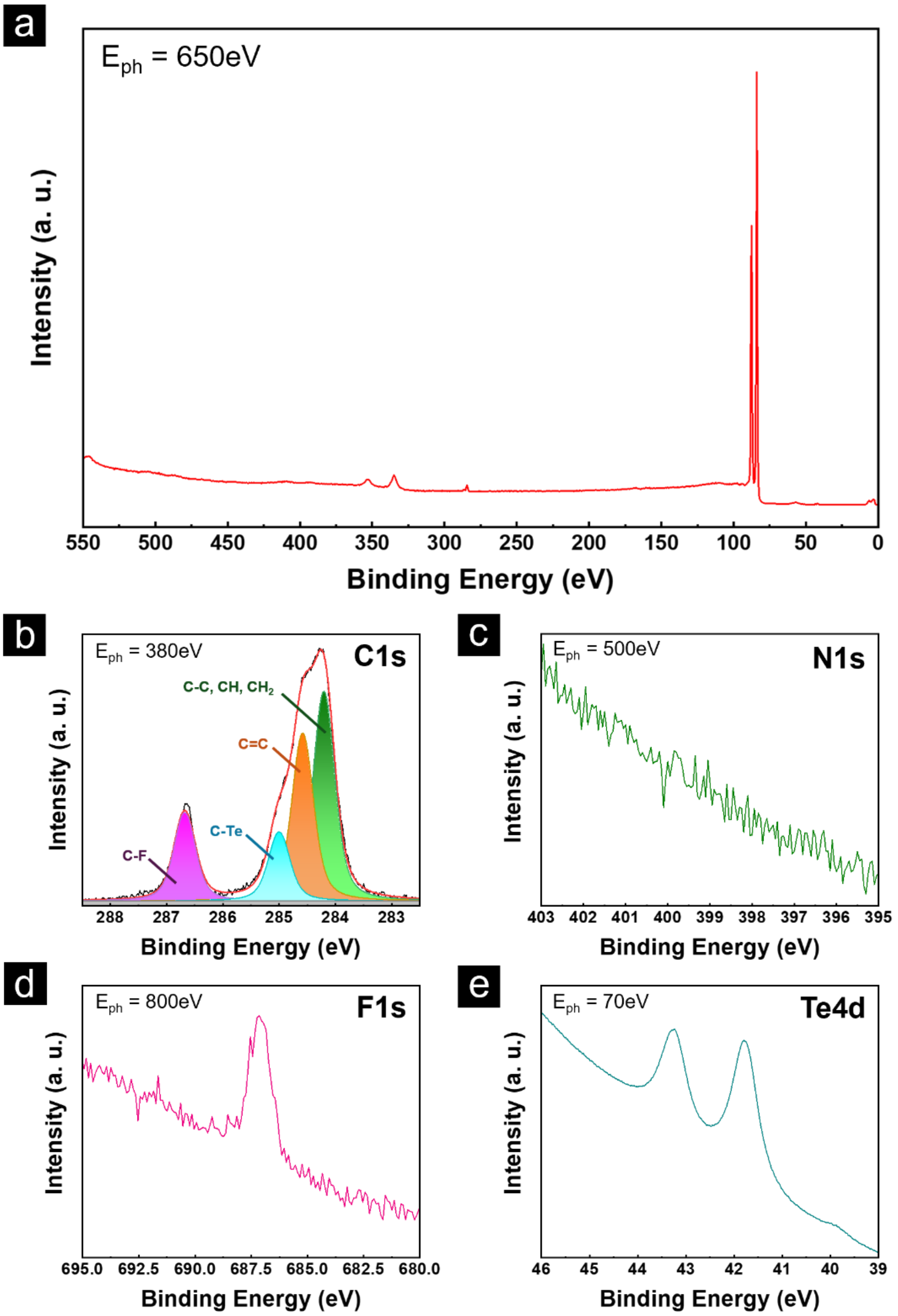

Figure S7 – XPS spectra of Au(111) sample after deposition of Benzo-3FBP-2Te molecule. (a) survey; (b) C1s core level fitted with four components indicated as C=C, (C-C, C-H, C-H_2), C-Te, C-F; (c) N1s core level showing a flat signal because nitrogen is missing; (d) F1s core level; (e) Te4d core level.

XPS data were acquired at the FlexPes beamline (20200269) at the MAX IV synchrotron radiation facility, tuning the photon energy as reported inside the figure panels, with a geometry configuration of the samples in normal emission with respect the analyser.

Survey spectra were acquired with a photon energy of 650eV, pass energy 100eV, slit 20; C1s spectra were acquired with a photon energy of 380eV, pass energy 20eV, slit 20; N1s spectra were acquired with a photon energy of 500eV, pass energy 20eV (50eV for Benzo-3FBP-2Te), slit 20; F1s spectra were acquired with a photon energy of 800eV, pass energy 20eV, slit 35; Te4d spectra were acquired with a photon energy of 70eV, pass energy 20eV, slit 20. All the parameters are the same for both 3FBP-2Te/Au(111) and Benzo-3FBP-2Te/Au(111) samples unless otherwise stated. The binding energy of each core level is calibrated after the acquisition of the Fermi level at the photon energy used to acquire the core level signal.

NMR characterization

2-butyl-1,3,4-trifluorobenzo[b]benzo[4,5]telluropheno[2,3-d]tellurophene

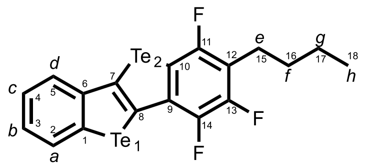

^{1}H NMR (600 MHz, $CDCl_3$) δ 8.03 (d, $J_{H,H}$ = 7.9 Hz, 1H, H_a), 7.68 (dd, $J_{H,H}$ = 7.9, 1.2 Hz, 1H, H_d), 7.47 – 7.42 (m, 1H, H_b), 7.23 (t, J = 7.5, 1.2 Hz, 1H, H_c), 2.81 – 2.76 (m, 2H, H_e), 1.69 – 1.61 (*m*, 2H, H_f), 1.41 (*m*, 2H, H_g), 0.96 (*t*, $J_{H,H}$ = 7.0 Hz, 3H, H_h).

^{19}F NMR (659 MHz, $CDCl_3$) δ -106.12 (*d*, $J_{F,F}$ = 16.5 Hz, 1F), -142.28(*dd*, $J_{F,F}$ = 18.9, 4.6 Hz, 1F), -144.00(*dd*, $J_{F,F}$ = 18.9, 16.5 Hz, 1F).

^{13}C NMR (151 MHz, $CDCl_3$) δ 155.94 (C_{13}), 148.73 (C_{11}), 146.06 (C_6), 143.62 (C_{14}), 135,21 (C_9), 133.17 (C_1), 132.73 (C_2), 131.82 (C_7), 127.94 (C_5), 126.47 (C_4), 125.49 (C_3), 121.51 (C_8), 116.24 (C_{12}), 109.60 (C_{10}), 31.77 (C_{15}), 23.10 (C_{16}), 22.54 (C_{17}), 13.94 (C_{18}).

^{125}Te NMR (190 MHz, $CDCl_3$) δ 877.51 (*d*, $^{4TS}J_{Te,F}$ = 290.4 Hz, Te_1), 819.40(*d*, $^{3}J_{Te,F}$ = 26.2 Hz, Te_2).

7-butyl-6,8,9-trifluorobenzo[4',5']telluropheno[2',3':4,5]telluropheno[2,3-β]pyridine 2.28

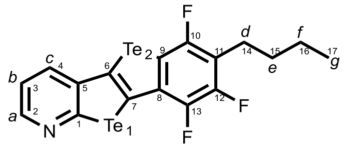

^{1}H NMR (600 MHz, CDCl3) δ 8.53 (dd, J = 4.7, 1.6 Hz, 1H, H_a), 7.86 (dd, J = 8.0, 1.6 Hz, 1H, H_c), 7.37 (dd, J = 7.9, 4.6 Hz, 1H, H_b), 2.79 (t, J = 7.7 Hz, 2H, H_d), 1.66 (p, J = 7.6 Hz, 2H, H_e), 1.45 – 1.39 (m, 2H, H_e), 0.96 (t, J = 7.4 Hz, 3H, H_g).

^{19}F NMR (659 MHz, $CDCl_3$) δ -105.33 (*dd*, $J_{F,F}$ = 16.8, 3.6 Hz, 1F), -141.71 (*dd*, $J_{F,F}$ = 20.0, 16.6 Hz, 1F), -142.44 (*dd*, $J_{F,F}$ = 20.0, 3.6 Hz, 1F).

^{13}C NMR (151 MHz, $CDCl_3$) δ 161.16 (C_1), 157.81 (C_{12}), 147.12 (C_{10}), 146.80 (C_3), 145.49 (C_{13}), 142.23 (C_5), 135.50 (C_8), 133.47 (C_4), 126.63 (C_6), 124.50 (C_7), 120.87 (C_2), 116.69 (C_{11}) 110.17 (C_9), 31.73 (C_{14}), 23.14 (C_{15}), 22.54 (C_{16}), 13.93 (C_{17}).

^{125}Te NMR (190 MHz, $CDCl_3$) δ 900.15 (*d*, $^{4TS}J_{Te,F}$ = 290.4 Hz, *Te_1*), 842.92 (*d*, $^{3}J_{Te,F}$ = 26.5 Hz, *Te_2*).

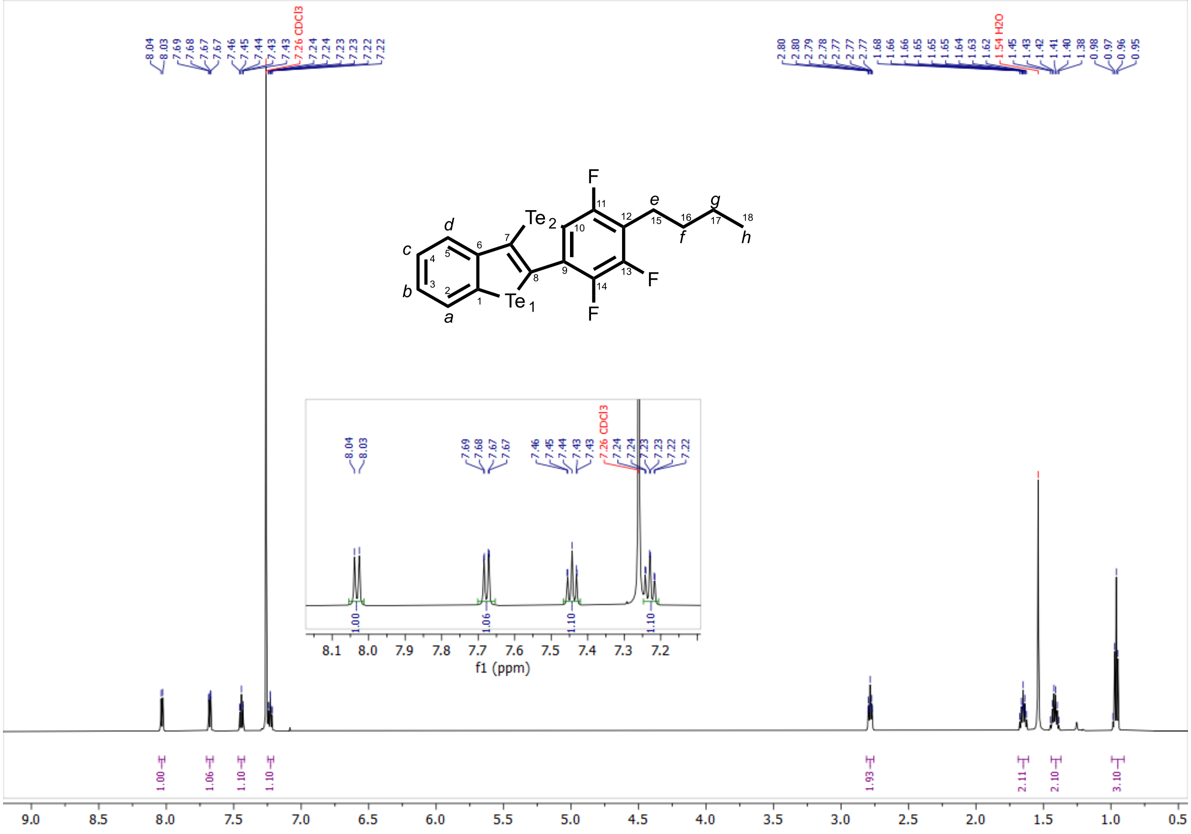

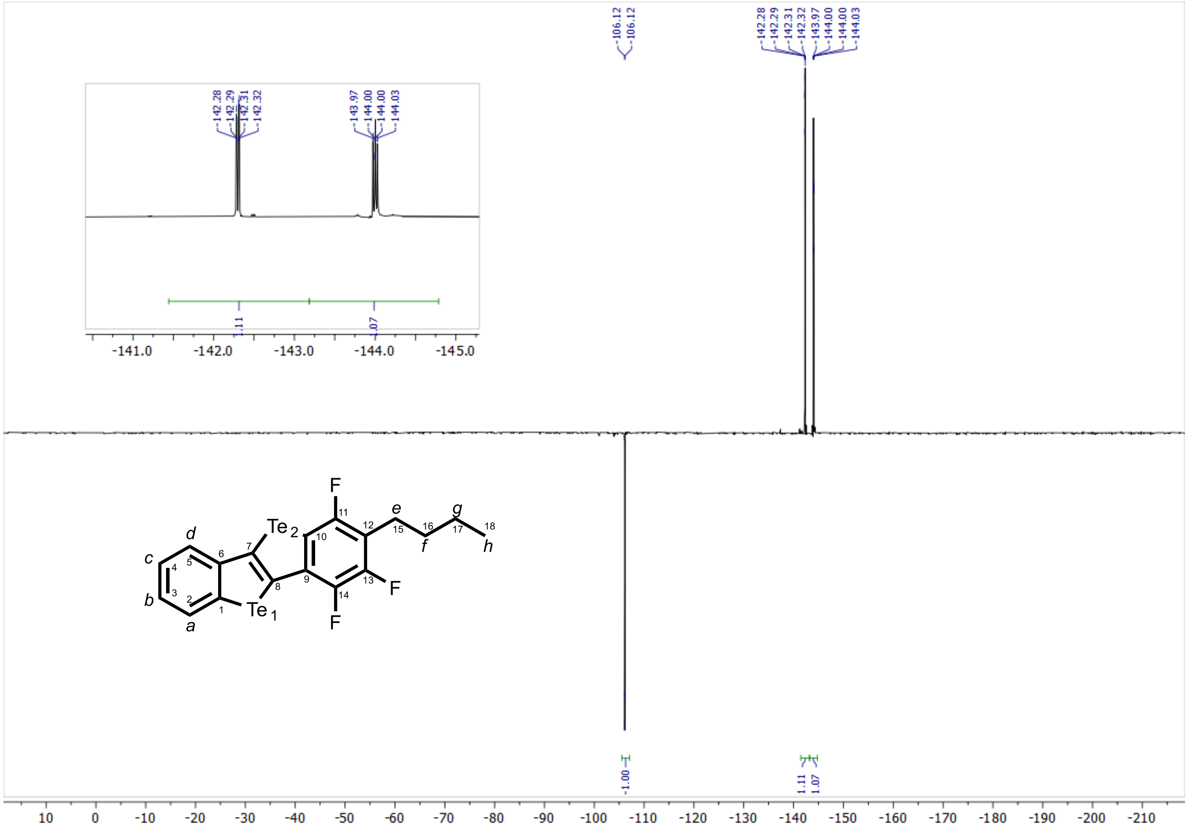

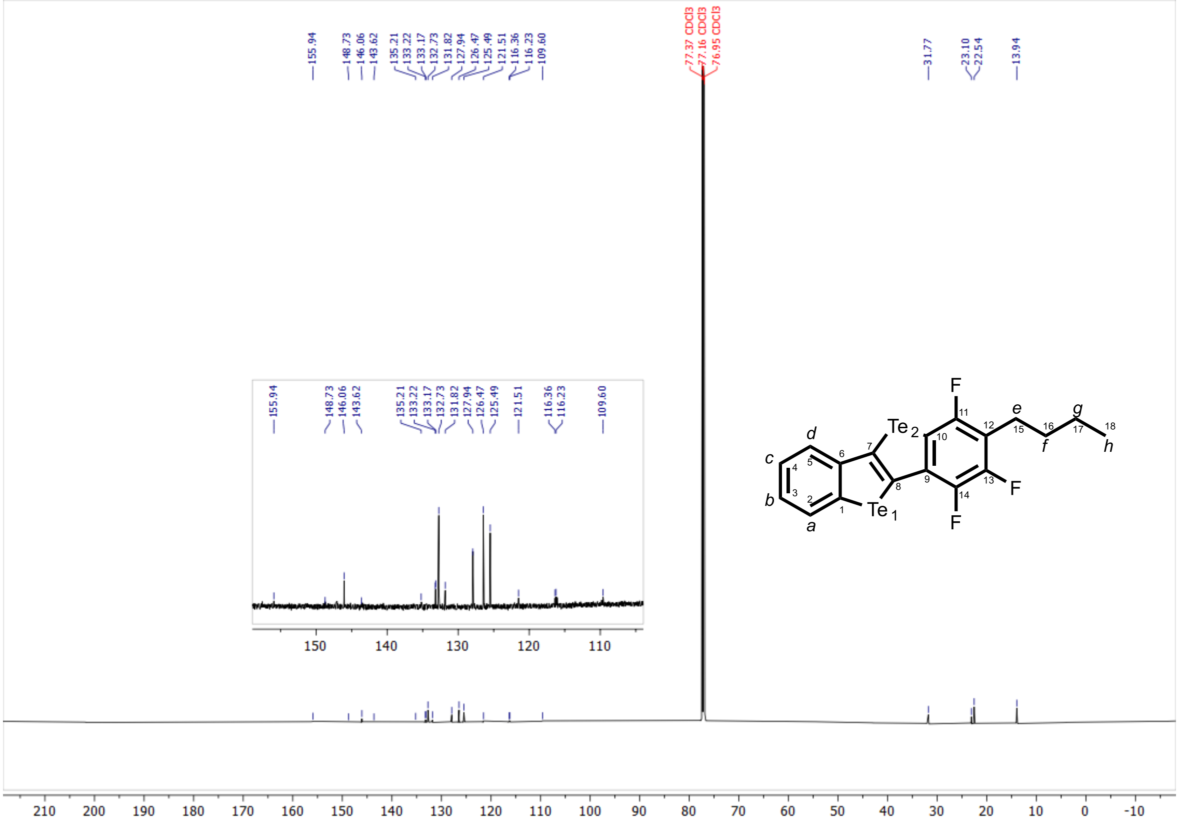
155.94
148.73
146.06
143.62
135.21
133.22
133.17
132.73
131.82
127.94
126.47
125.49
121.51
116.36
116.23
109.60
77.37 CDCl3
77.16 CDCl3
76.95 CDCl3
31.77
23.10
22.54
13.94
Te 2
Te 1
F
F
F
e
f
g
h
a
b
c
d
210
200
190
180
170
160
150
140
130
120
110
100
90
80
70
60
50
40
30
20
10
0
-10

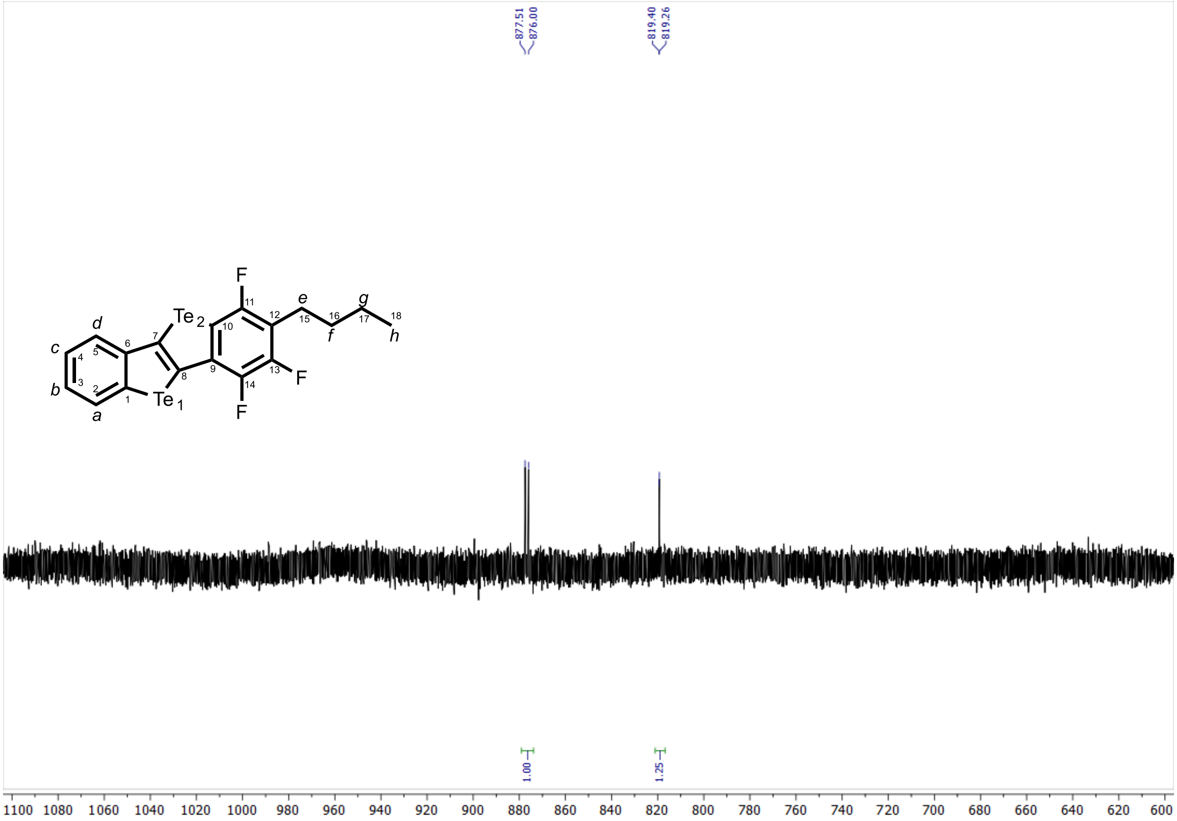
877.51
876.00
819.40
819.26
Te 2
Te 1
F
F
F
e
f
g
h
a
b
c
d
1.00
1.25
1100
1080
1060
1040
1020
1000
980
960
940
920
900
880
860
840
820
800
780
760
740
720
700
680
660
640
620
600

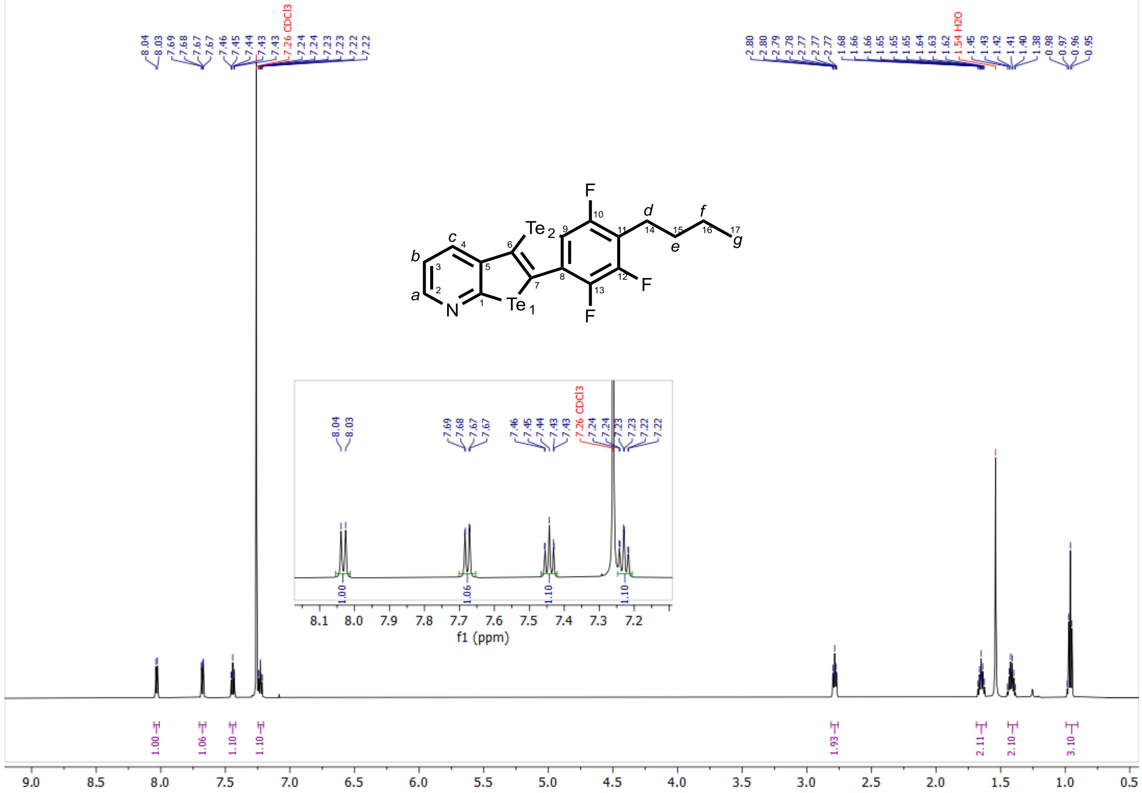

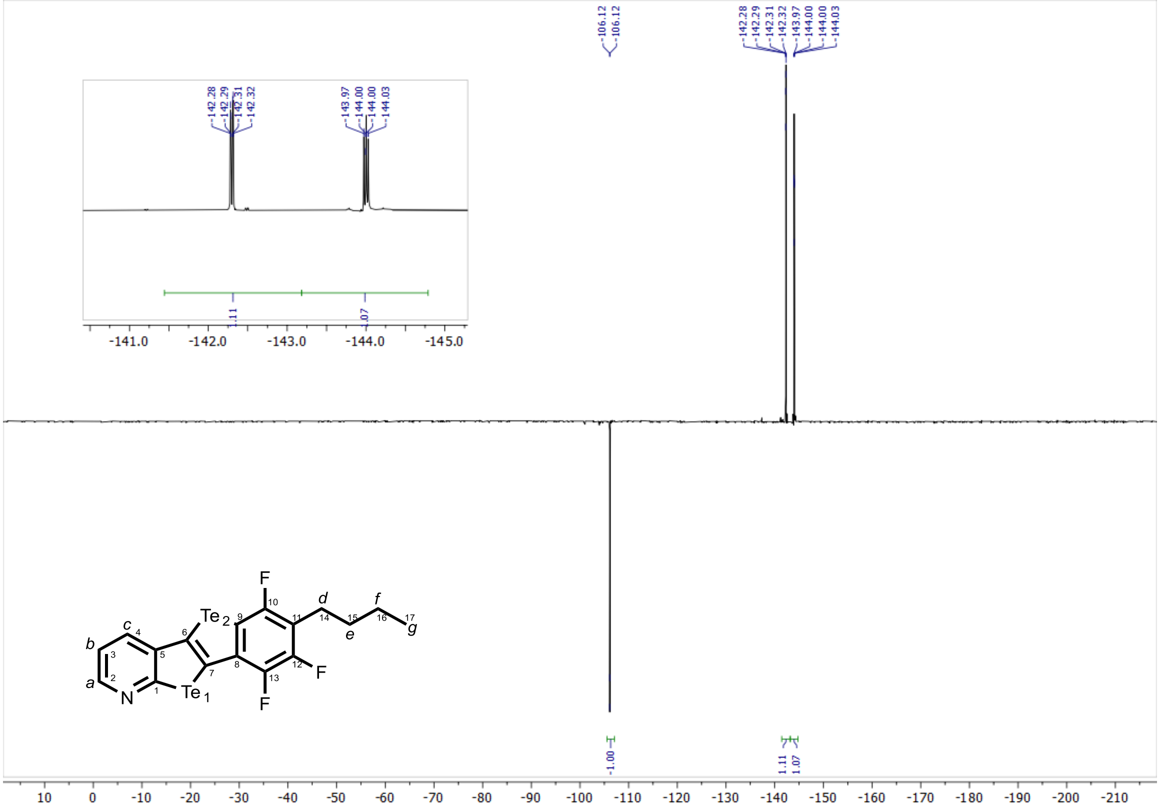

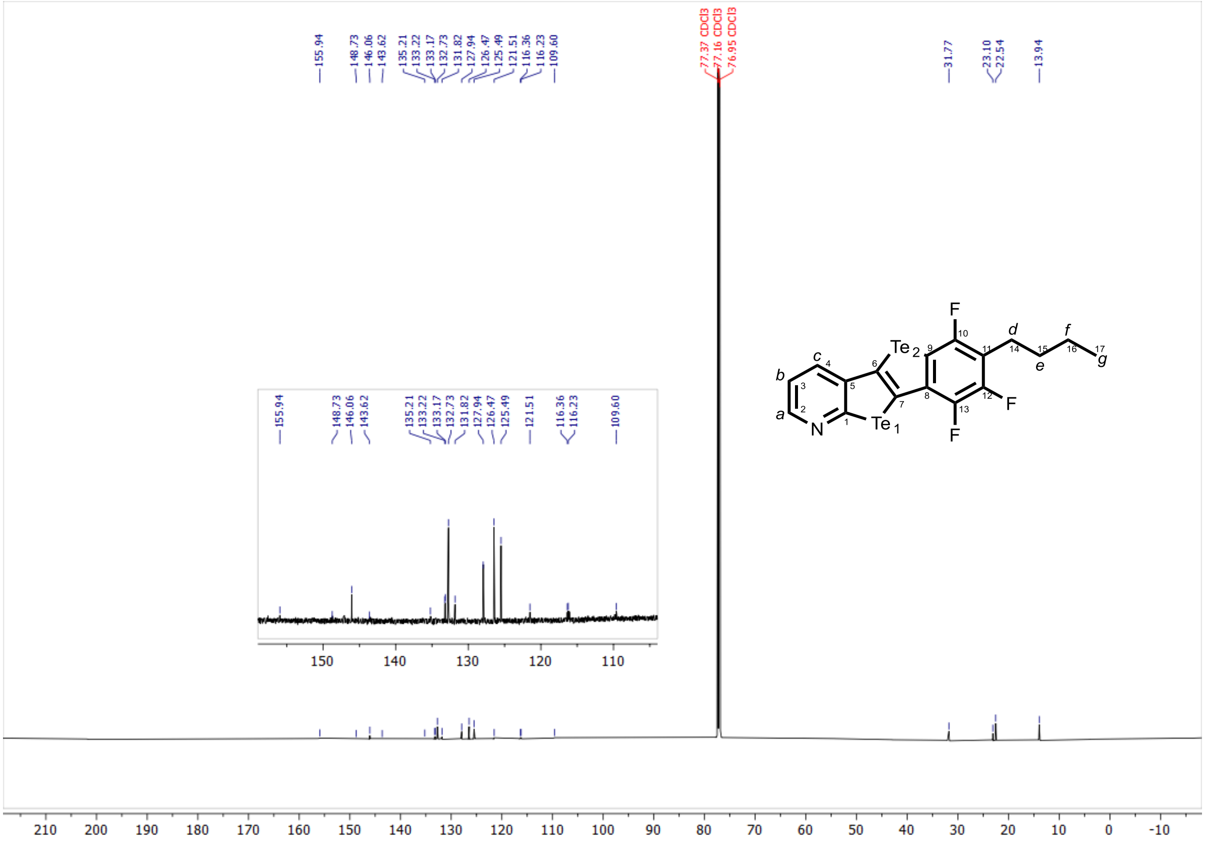

155.94
148.73
146.06
143.62
135.21
133.22
133.17
132.73
131.82
127.94
126.47
125.49
121.51
116.36
116.23
109.60
77.37 CDCl3
77.16 CDCl3
76.95 CDCl3
31.77
23.10
22.54
13.94
F
Te 2
Te 1
N
F
F
a
b
c
d
e
f
g
210 200 190 180 170 160 150 140 130 120 110 100 90 80 70 60 50 40 30 20 10 0 -10
150 140 130 120 110

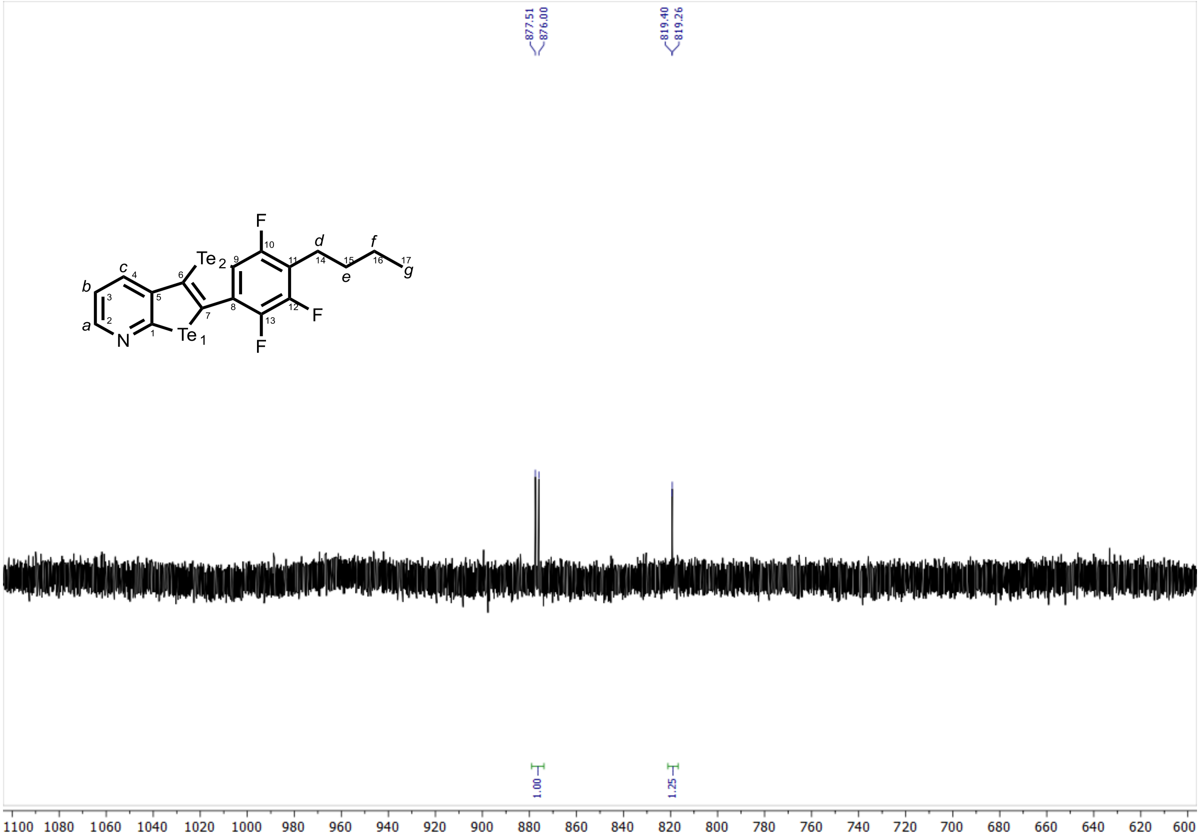

877.51
876.00
819.40
819.26
F
Te 2
Te 1
N
F
F
a
b
c
d
e
f
g
1.00
1.25
1100 1080 1060 1040 1020 1000 980 960 940 920 900 880 860 840 820 800 780 760 740 720 700 680 660 640 620 600

REFERENCES

1. P. Giannozzi, S. Baroni, N. Bonini, M. Calandra, R. Car, C. Cavazzoni, D. Ceresoli, G. L. Chiarotti, M. Cococcioni, I. Dabo, A. Dal Corso, S. de Gironcoli, S. Fabris, G. Fratesi, R. Gebauer, U. Gerstmann, C. Gougoussis, A. Kokalj, M. Lazzeri, L. Martin-Samos, N. Marzari, F. Mauri, R. Mazzarello, S. Paolini, A. Pasquarello, L. Paulatto, C. Sbraccia, S. Scandolo, G. Sclauzero, A. P. Seitsonen, A. Smogunov, P. Umari and R. M. Wentzcovitch, QUANTUM ESPRESSO: a modular and open-source software project for quantum simulations of materials, *Journal of Physics: Condensed Matter*, 2009, **21**, 395502.
2. J. P. Perdew, K. Burke and M. Ernzerhof, Generalized Gradient Approximation Made Simple, *Physical Review Letters*, 1996, **77**, 3865-3868.
3. S. Grimme, S. Ehrlich and L. Goerigk, Effect of the damping function in dispersion corrected density functional theory, *Journal of Computational Chemistry*, 2011, **32**, 1456-1465.
4. L. Camilli, C. Hogan, D. Romito, L. Persichetti, A. Caporale, M. Palummo, M. Di Giovannantonio and D. Bonifazi, On-Surface Molecular Recognition Driven by Chalcogen Bonding, *JACS Au*, 2024, **4**, 2115-2121.
5. K. Momma and F. Izumi, VESTA 3 for three-dimensional visualization of crystal, volumetric and morphology data, *Journal of Applied Crystallography*, 2011, **44**, 1272-1276.
6. E. R. Johnson, S. Keinan, P. Mori-Sánchez, J. Contreras-García, A. J. Cohen and W. Yang, Revealing Noncovalent Interactions, *Journal of the American Chemical Society*, 2010, **132**, 6498-6506.
7. J. Tersoff and D. R. Hamann, Theory of the scanning tunneling microscope, *Physical Review B*, 1985, **31**, 805-813.
